# Body Mass Index and Postsurgical Outcomes in Older Adults

**DOI:** 10.1001/jamanetworkopen.2025.28875

**Published:** 2025-08-26

**Authors:** Cecilia Canales, Myles Anderson, David Elashoff, Tristan Grogan, Marcia M. Russell, Victor Duval, Robert Whittington, Maxime Cannesson, Catherine Sarkisian

**Affiliations:** 1 Department of Anesthesiology and Perioperative Medicine, David Geffen School of Medicine at University of California, Los Angeles; 2 Charles R. Drew University of Medicine and Science, Los Angeles, California; 3 Division of General Internal Medicine and Health Services Research, Department of Medicine, David Geffen School of Medicine at University of California, Los Angeles; 4 Department of Medicine Statistics Core, David Geffen School of Medicine at University of California, Los Angeles; 5 Department of Surgery, David Geffen School of Medicine at University of California, Los Angeles; 6 VA Greater Los Angeles Healthcare System, Surgical and Perioperative Careline, Los Angeles, California; 7 VA Greater Los Angeles Healthcare System, Geriatrics Research Education and Clinical Center, Los Angeles, California

## Abstract

**Importance:**

High body mass index (BMI) has been associated with increased postoperative complications including mortality in the general population, leading many perioperative clinicians to recommend preoperative lifestyle modifications aimed at achieving normal body weight. However, aging introduces physiological changes associated with frailty, such as altered body composition, fat redistribution, and stature reduction due to height loss, all of which may modify the association between BMI and surgical outcomes in older adults.

**Objective:**

To determine if a higher BMI in older adults who are undergoing major elective surgery is associated with rates of all-cause mortality.

**Design, Setting, and Participants:**

Cohort study of adults aged 65 years or older presenting for surgery from February 2019 to January 2022 at a preoperative clinic before planned major elective surgery at a large academic Center in Southern California.

**Exposure:**

Body mass index.

**Main Outcomes and Measures:**

Postoperative outcomes included all-cause 30-day and 1-year mortality, postoperative delirium, discharge disposition, and complications classified using the Clavien-Dindo system.

**Results:**

The study included 414 older adults undergoing major elective surgery with a mean (SD) age of 75.9 (7.2) years; 54.8% (95% CI, 50.2%-60.4%) of the cohort were female. The prevalence of frailty was 24.2% (95% CI, 20.3%-28.5%), and 37.0% (95% CI, 32.6%-41.8%) of the cohort was prefrail. The overall 30-day all-cause mortality rate was 11.0% (95% CI, 8.5%-14.5%). Patients categorized as overweight (BMI, 25.0-29.9; calculated as weight in kilograms divided by height in meters squared) had the lowest 30-day all-cause mortality rate, with a significant risk reduction compared with patients with a normal BMI (18.5-24.9) (1 of 128 patients [0.8%] vs 25 of 133 patients [18.8%]; odds ratio [OR], 0.03; 95% CI, 0.01-0.26; *P* = .001). This association remained significant in the multivariable logistic regression model after adjusting for potential confounders (OR, 0.14; 95% CI, 0.06-0.34; *P* < .001). Patients categorized as underweight (BMI <18.5) had the highest 30-day all-cause mortality rate (15 of 20 patients [75.0%]; 95% CI, 55.0%-90.0%).

**Conclusions and Relevance:**

In this observational cohort study of older adults undergoing major elective surgery, being overweight was associated with lower odds of 30-day all-cause mortality. These findings suggest that traditional weight loss recommendations based on achieving normal BMI may need to be reevaluated for this population.

## Key Points

**Question:**

In older adults (≥65 years) presenting for surgery, is higher body mass index associated with lower 30-day all-cause mortality?

**Findings:**

In this cohort study of 414 participants, patients categorized as being overweight had the lowest 30-day all-cause mortality rate compared with patients categorized as having a normal body mass index.

**Meaning:**

These findings suggest traditional body mass index risk categories may need to be recalibrated for the population aged 65 years and older undergoing surgery.

## Teaser

This cohort study evaluates whether higher body mass index in older adults who are undergoing major elective surgery is associated with rates of all-cause mortality.

## Introduction

More than half of US residents are classified as overweight or obese based on their body mass index (BMI), with a growing number of adults aged 65 years and older falling into the overweight and obese categories.^1^ Higher BMI is strongly associated with an increased risk of chronic conditions such as cardiovascular disease, type 2 diabetes, obstructive sleep apnea, and certain cancers, as well as decreased life expectancy in the general population.^2,3,4,5^ BMI, calculated as weight in kilograms divided by height in meters squared, classifies individuals into categories such as underweight (<18.5), normal weight (18.5-24.9), overweight (25.0-29.9), obese (30.0-34.9), and morbidly obese (≥35.0).^6^ Population-based studies in younger and middle-aged adults often demonstrate a J-shaped relationship between BMI and outcomes, with the lowest risk of all-cause mortality observed among those with normal BMI and increased mortality at the extremes of BMI categories.^7,8,9^

However, body composition and its implications for health outcomes vary substantially by age, gender, and race.^10,11^ Aging is associated with functional decline, multimorbidity, and frailty, which independently contribute to poorer postoperative outcomes.^10,12^ Emerging evidence suggests that in community-dwelling older adults, a higher BMI may be protective against all-cause mortality.^7,9^ This protection has been hypothesized to stem from increased physiological reserve, better nutritional status, or greater metabolic adaptability, which may provide resilience against age-related health declines and acute stressors.

In the perioperative setting across patients of all ages, high BMI has been linked to increased postoperative complications, including blood loss, infection, and thromboembolic events.^13,14^ Additionally, higher BMI has been linked to greater postoperative mortality in certain populations.^15^ As a result, many perioperative clinicians recommend preoperative lifestyle modifications, including weight loss, with the intention of optimizing surgical outcomes and reducing obesity-related health risks.^3,16,17^

Despite these efforts, the relationship between BMI and perioperative outcomes remains unclear in older adults. Given that older adults experience unique age-related physiological changes which may modify the impact of BMI on health outcomes,^10,12^ questions remain about the appropriateness of current recommendations for overweight older adults to lose weight before surgery.^17^ We hypothesized that higher BMI in older adults undergoing major elective surgery would be associated with lower risk of all-cause mortality. To test whether higher BMI is associated with better survival rates for older adults undergoing major elective surgery, we conducted a longitudinal cohort study of older adults undergoing major elective surgery at a large urban academic center.

## Methods

After institutional review board approval, waiver of consent due to use of deidentified data, and in accordance with the Strengthening the Reporting of Observational Studies in Epidemiology (STROBE) reporting guidelines,^18^ we conducted a cohort study of older adults presenting to our preoperative clinic at a large academic center in southern California. The process for preoperative evaluation at University of California at Los Angeles Health has been described in detail elsewhere.^19^ Briefly, the preoperative clinic evaluates over 36 000 patients annually. Those deemed appropriate for a telephone preoperative interview are assessed in that manner, with the remaining approximately 1200 patients evaluated in the clinic annually. All adults aged 65 years or older who present for an in-person assessment in the preoperative clinic undergo routine standardized geriatric and frailty assessments.

### Study Population

In this study we included all adults aged 65 years or older who were assessed by the preoperative clinic for noncardiac, nontransplant, elective surgery and expected to require postoperative hospitalization of at least 1 night over a 3-year period (February 2019 to January 2022). Patients scheduled for intracranial neurosurgery were excluded as well. Those patients who presented to the preoperative clinic multiple times for medical optimization or for multiple surgical procedures were only included during their last assessment before surgery. The last visit was chosen, as weight may vary from the first to last visit. Therefore, the cohort was composed of only unique patient visits.

### Demographic and Frailty Assessments

In addition to measuring height and weight to account for potential moderating or confounding factors, we collected demographic and clinical data. This included age, gender, self-reported race (collected as standard patient care; Asian, Black/African American, Hispanic, White, other, or not disclosed) height, weight, nutrition and weight loss screening results, American Society of Anesthesiologists (ASA) status, type of surgery, and comorbidities such as hypertension and diabetes. All data including height, weight, and comorbidities were recorded by the preoperative clinic nursing team during the preoperative clinic encounters. Patients were categorized based on BMI into groups: underweight (<18.5), normal weight (18.5-24.9), overweight (25.0-29.9), obese (30.0-39.9), and morbidly obese (>40.0). Given that cancer is associated with unintentional weight loss and overall outcomes (including mortality), we also specifically noted if a patient had an active cancer diagnosis at the time of the preoperative visit. Frailty was assessed using the 5-item modified frailty index (mFI-5) prospectively by a trained nurse at the time of the patient’s preoperative clinic visit.^20^ The modified frailty scale uses health deficit accumulation and includes comorbid factors of (1) congestive heart failure (within 30 days of surgery), (2) type 2 diabetes, (3) chronic obstructive pulmonary disease or pneumonia; (4) dependent functional health status (total or partial) at time of surgery; and (5) hypertension requiring medication.

### Postsurgical Outcomes Data

The primary outcomes were 30-day and 1-year postoperative all-cause mortality. Our secondary outcome was the incidence of complications. We (C.C. and M.A.) performed a review of each patient’s electronic medical record to obtain postsurgical complications, nonhome discharge disposition, and all-cause 30-day and 1-year postoperative mortality. The mFI-5 was calculated based on the sum of categories and assigned into frail (mFI-5 ≥2); prefrail (mFI-5 1); or not frail (mFI-5 0). Complications were categorized as reoperation, readmission within 30 days, in-hospital cardiac events (ie, arrhythmia or myocardial infarction), in-hospital pulmonary events (ie, reintubation or in-hospital pneumonia), and other in-hospital complications including surgical site infection, deep vein thrombosis (DVTs), and strokes. We also calculated the Clavien-Dindo complication classification, as it offers a standardized method for comparing severity of complications across diverse surgical populations at 30 days postoperatively.^21^ Delirium was identified in the first 5 days after surgery both by assessment with the Confusion Assessment Method (CAM) and medical record review using published criteria.^22^ Hospitalized patients aged 65 years or older at our institution undergo delirium screening using CAM or CAM-ICU (intensive care unit) twice per day by nursing staff as standard patient care (every 12-hour shift). CAM scores are entered into the electronic medical record. We elected a priori to also include delirium by medical record review, as it has been shown to complement CAM, given that delirium is a state of altered consciousness that waxes and wanes throughout the day.^23^ CAM assessments can miss acute episodes of delirium.

### Statistical Analysis

The primary independent variable of interest was BMI. Patient characteristics and study variables were summarized by BMI groups using mean (SD) or frequency (percentage) unless otherwise noted and compared between groups using the 1-way analysis of variance or χ^2^ test. The primary outcomes were 30-day and 1-year all-cause mortality. Secondary outcomes included the incidence of complications and specifically severe complications (Clavien-Dindo Complication grade 3 or higher) defined as any complication leading to invasive intervention, reoperation, unplanned ICU admission, single or multiorgan failure, or death within 30 days of surgery.^21^ To identify an optimal BMI cutoff associated with mortality, we applied the Youden index, a commonly used metric in diagnostic testing. The Youden index is calculated as sensitivity + specificity – 1 and identifies the point on the receiving operating characteristic curve that maximizes the combined sensitivity and specificity of a given cutoff. However, since we hypothesized having low or high BMI were associated with mortality, we conducted all further analyses using standard BMI categories for consistency and interpretability. To evaluate the association between BMI categories and mortality, multivariable logistic regression models were used to calculate adjusted odds ratios (ORs) with 95% CIs. Normal BMI (18.5–24.9) was used as the reference group based on prior population studies indicating the lowest all-cause mortality in this category. Adjustments were made for key potential confounders, including age, sex, ASA status, and frailty, to account for their potential influence on the outcomes. All tests were 2-tailed, and a *P* value less than .05 was considered statistically significant. All statistical analyses were performed using SPSS version 29 (IBM) or R version 4.1.0 (R Project for Statistical Computing).

A power analysis was conducted to assess the minimal detectable ORs for each BMI category (<18.5, 18.5-24.9, 25.0-29.9, 30.0-39.9, and >40.0) in relation to the 2 outcomes (30-day and 1-year mortality). The sample sizes varied by BMI group, ranging from 20 patients in the less than 18.5 group to 133 in the normal BMI group. For 30-day mortality, the minimal detectable ORs were 2.16, 0.34, 0.31, and 0.004, respectively. For 1-year mortality, the minimal detectable ORs were 3.72, 0.43, 0.40, and 0.07, respectively. These estimates were derived using the observed sample sizes and mortality rates and reflect the smallest effect sizes that could be detected with 80% power at a significance level of .05. Given these values, the study was adequately powered to detect clinically meaningful differences in outcomes across BMI groups.

## Results

**Figure 1.**
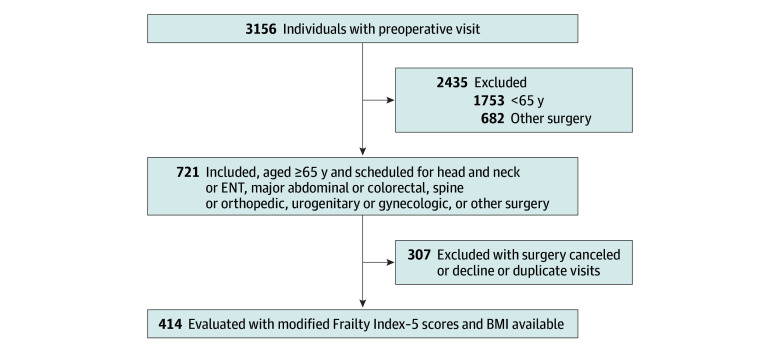
Study Flow Diagram Depicts study population, inclusion, exclusion, surgical cases canceled, and final study population. BMI indicates body mass index; ENT, otolaryngology.

Over a 3-year period, we enrolled 414 consecutive older adults with a mean (SD) age of 75.9 ( 7.20) years; women represented 54.8% (95% CI, 50.2%-60.4%) (Figure 1). Baseline characteristics (Table 1) showed no statistical differences in demographics, ASA status, and surgical type between the BMI groups. The prevalence of frailty was 24.2% (95% CI, 20.3%-28.5%), while 37.0% were prefrail (95% CI, 32.6%-41.8%) (Table 2).

**Table 1.** Baseline Characteristics by Body Mass Index

Characteristic	Participants, No. (%)						*P* value
	Total (n = 414)	Body mass index categories^a^					
		<18.5 (n = 20)	18.5-24.9 (n = 133)	25.0-29.9 (n = 128)	30.0-39.9 (n = 109)	40.0>(n = 24)	
Age, mean (SD), y	75.9 (7.2)	74.2 (7.9)	75.9 (7.2)	76.3 (7.0)	76.4 (7.4)	73.1 (6.0)	.23
Sex							
Female	227 (54.8)	14 (70.0)	71 (53.4)	63 (49.2)	64 (58.7)	15 (62.5)	.31
Male	187 (45.2)	6 (30.0)	62 (46.6)	65 (50.7)	45 (41.3)	9 (37.5)	
ASA status							
2	93 (22.5)	1 (5.0)	35 (26.3)	28 (21.9)	24 (22.0)	5 (20.8)	.19^b^
3	285 (68.8)	19 (95.0)	84 (63.2)	89 (69.5)	78 (71.6)	15 (62.5)	
4	36 (8.7)	0	14 (10.5)	11 (8.6)	7 (6.4)	4 (16.7)	
Race and ethnicity^c^							
Asian	34 (8.2)	1 (5)	16 (12)	10 (7.8)	7 (6.4)	0	.42^b^
Black/African American	27 (6.5)	1 (5)	12 (9)	7 (5.5)	5 (4.6%	2 (8.3)	
Hispanic	40 (9.7)	1 (5)	10 (7.5)	12 (9.4)	13 (11.9)	4 (16.4)	
White	300 (72.5)	16 (80)	93 (69.9)	91 (71.1)	82 (75.2)	18 (75)	
Other/not disclosed	13 (3.1)	1 (5)	2 (1.5)	8 (6.3)	2 (1.8)	0	
Surgical type							
Head and neck/otolaryngology	83 (20.0)	6 (30.0)	26 (19.5)	27 (21.1)	18 (16.5)	6 (25.0)	.70^b^
Major abdominal/colorectal	69 (16.7)	4 (20.0)	25 (18.8)	20 (15.6)	19 (17.4)	1 (4.2)	
Other	38 (9.2)	2 (10.0)	14 (10.5)	7 (5.5)	13 (11.9)	2 (8.3)	
Spine/orthopedic	133 (32.1)	4 (20.0)	36 (27.1)	47 (36.7)	37 (33.9)	9 (37.5)	
Urology/gynecology	91 (22.0)	4 (20.0)	32 (24.1)	27 (21.1)	22 (20.2)	6 (25.0)	
Cancer	179 (43.2)	9 (45.0)	57 (42.9)	51 (39.8)	53 (48.6)	9 (37.5)	.69
Frail status							
Frail	100 (24.2)	4 (20.0)	31 (23.3)	31 (24.2)	23 (21.1)	11 (45.8)	.11^b^
Prefrail	153 (37.0)	9 (45.0)	41 (30.8)	56 (43.8)	40 (36.7)	7 (29.2)	
Not frail	161 (38.9)	7 (35.0)	61 (45.9)	41 (32.0)	46 (42.2)	6 (25.0)	
Recommended weight loss	107 (25.8)	0	8 (6.0)	21 (16.4)	54 (49.5)	24 (100.0)	<.001^b^
Unintentional weight loss	44 (10.6)	9 (45.0)	19 (14.3)	12 (9.4)	4 (3.7)	0	<.001^b^

Abbreviation: ASA, American Society of Anesthesia.

^a^ Body mass index calculated as weight in kilograms divided by height in meters squared.

^b^ Fisher exact test.

^c^ Race and ethnicity was self-reported; other/not disclosed was its own category without detailed subcategories.

**Table 2.** Logistic Regression Models

Characteristic	30-d Mortality outcome				1-y Mortality outcome			
	Univariable models		Multivariable model		Univariable models		Multivariable model	
	OR (95% CI)	*P* value	OR (95% CI)	*P* value	OR (95% CI)	*P* value	OR (95% CI)	*P* value
BMI category^a^								
<18.5	12.96 (4.31-39.00)	<.001	21.81 (6.24-76.21)	<.001	10.00 (3.14-31.85)	<.001	12.40 (3.70-41.61)	<.001
25.0-29.9	0.03 (0.01-0.26)	.001	0.03 (0.00-0.23)	.001	0.14 (0.06-0.34)	<.001	0.13 (0.06-0.32)	<.001
30.0-39.9	0.04 (0.01-0.30)	.002	0.03 (0.00-0.24)	.001	0.12 (0.05-0.32)	<.001	0.10 (0.04-0.28)	<.001
>40.0	1.14 (0.39-3.34)	.82	0.82 (0.24-2.79)	.75	0.83 (0.31-2.26)	.72	0.62 (0.21-1.83)	.39
Frailty								
Prefrail vs not frail	0.63 (0.28-1.44)	.28	0.43 (0.15-1.26)	.13	0.73 (0.38-1.39)	.33	0.66 (0.30-1.44)	.30
Frail vs not frail	2.41 (1.19-4.88)	.02	3.19 (1.24-8.20)	.05	2.22 (1.21-4.08)	.01	2.69 (1.27-5.72)	.01
Age	NA	NA	1.05 (1.00-1.11)	.07	NA	NA	1.03 (0.98-1.07)	.22
Female	NA	NA	1.64 (0.74-3.60)	.22	NA	NA	1.53 (0.83-2.83)	.17
ASA	NA	NA	1.72 (0.85-3.48)	.13	NA	NA	1.30 (0.75-2.26)	.35

Abbreviations: ASA, American Society of Anesthesiologists; BMI, body mass index; NA, not applicable; OR, odds ratio.

^a^ BMI reference group was BMI between 18.5 and 24.9.

### Primary Outcome

**Figure 2.**
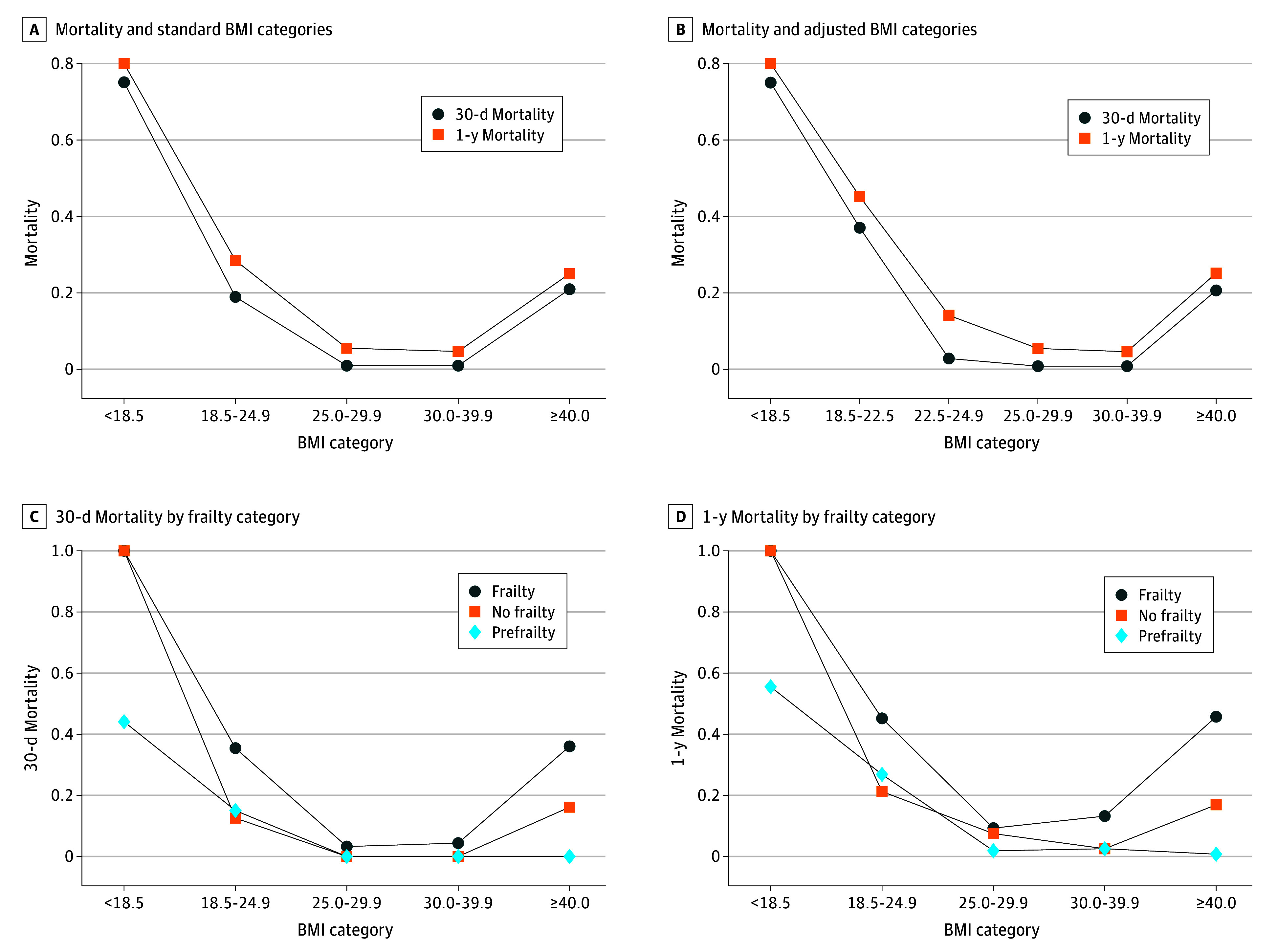
All-Cause Mortality at 30 Days and 1 Year A, Thirty-day and 1-year mortality based on standard body mass index (BMI; calculated as weight in kilograms divided by height in meters squared) categories. B, Thirty-day and 1-year mortality based on adjusted BMI categories from the Youden index cut-off at BMI of 22.5. C, 30-Day mortality by frailty status based on standard BMI categories. D, One-year mortality by frailty status based on standard BMI categories.

Overall, 11.0% (95% CI, 8.5%-14.5%) of patients experienced mortality at 30 days, and 17.0% (95% CI, 13.7%-21.3%) at 1 year. Univariable logistic regression showed that underweight patients had significantly higher odds of 30-day mortality compared with normal weight patients (OR, 12.9; 95% CI, 4.3-38.9; *P* < .001). In contrast, patients with overweight and obesity demonstrated a significantly lower 30-day mortality risk compared with normal weight patients (overweight OR, 0.03; 95% CI, 0.01-0.26; *P* = .001; obese OR, 0.04; 95% CI, 0.01-0.30; *P* = .02) for patients with obesity. Similarly, underweight patients had a significantly higher risk of 1-year mortality compared with normal weight patients (OR, 10.0; 95% CI, 3.1-31.8; *P* < .001), while patients with overweight (OR, 0.14; 95% CI, 0.06-0.34; *P* = .001) and obesity (OR, 0.12; 95% CI, 0.05-0.32; *P* = .02) showed lower risk. The multivariable models yielded similar findings (Table 3). Using the Youden index, a BMI cutoff of 22.5 was identified. As a sensitivity and hypothesis generating subanalysis, we further subdivided the normal weight category into 18.5 to 22.5 and 22.5 to 25 (Figure 2; eTable 1 in Supplement 1).

**Table 3.** Complications by Body Mass Index and Frailty

Characteristic	Body mass index categories^a^						Frailty status			
	<18.5 (n = 20)	18.5-24.9 (n = 133)	25.0-29.9 (n = 128)	30.0-39.9 (n = 109)	40.0>(n = 24)	*P* value^b^	Not frail (n = 161)	Prefrail (n = 153)	Frail (n = 100)	*P* value
30 d Mortality	15 (75.0)	25 (18.8)	1 (0.8)	1 (0.9)	5 (20.8)	<.001	16 (9.9)	10 (6.5)	21 (21.0)	.001
1-y Mortality	16 (80.0)	38 (28.6)	7 (5.5)	5 (4.6)	6 (25.0)	<.001	25 (15.5)	18 (11.8)	29 (29.0)	.001
Delirium	10 (50.0)	19 (14.3)	14 (10.9)	14 (12.8)	5 (20.8)	.001	18 (11.2)	22 (14.4)	22 (22.0)	.06
Discharge other than home	10 (50.0)	21 (15.8)	9 (7.0)	16 (14.7)	7 (29.2)	<.001	18 (11.2)	23 (15.0)	22 (22.0)	.06
Clavien-Dindo classification										
3	0	1 (0.8)	0	1 (0.9)	1 (4.2)	<.001	2 (1.2)	1 (0.7)	0	<.001^a^
4	0	3 (2.3)	0	0	2 (8.3)		2 (1.2)	0	3 (3.0)	
5	11 (55.0)	20 (15.0)	1 (0.8)	1 (0.9)	4 (16.7)		12 (7.5)	8 (5.2)	17 (17.0)	

^a^ Body mass index calculated as weight in kilograms divided by height in meters squared.

^b^ Fisher exact test.

### Secondary Outcomes

Both extremes of BMI categories, underweight and morbidly obese, were associated with higher risk of postoperative complications (Table 3; eTable 2 and 3 in Supplement 1). In unadjusted analyses, underweight patients experienced postoperative delirium in 50.0% (95% CI, 35.0%-65.0%) of cases, and patients with morbid obesity experienced postoperative delirium in 21.0% (95% CI, 8.3%-33.3%) of cases (combined extremes rate of 34.1%; 95% CI, 22.7%-45.5%), compared with patients with normal weight, overweight, and obesity who experienced postoperative delirium in 12.7% of cases (95% CI, 9.7%-15.9%; *P* < .001). Patients with BMIs higher than 40.0 had the highest complication rate, with 79% experiencing at least 1 complication. When comparing types of complications, patients with morbid obesity were at greatest risk of pulmonary complications (33.0%; 95% CI, 20.8%-45.8%) and were more likely to have other complications including DVTs and strokes (21.0%; 95% CI, 8.3%-33.3%). Overweight patients had the fewest number of postoperative complications, with just 16.0% (95% CI, 10.2%-22.7%) experiencing any complication. When comparing by frailty status, frail patients were at highest risk of complication with 20.0% (95% CI, 14.0%-26.0%) of frail patients experiencing severe Clavien-Dindo complications (grade 3 or higher) (eTable 4 in Supplement 1).

## Discussion

Our study findings challenge the traditional belief that attaining a normal BMI (18.5–24.9) is ideal before major elective surgery for older adults. Instead, our findings suggest that older adults, particularly frail individuals, may benefit from being in the overweight category. Specifically, patients with a BMI of 25.0 to 29.9 had higher odds of survival at 30 days and 1 year following surgery compared with those with normal body weight. This BMI range was also associated with fewer overall postoperative complications, and these patients were more likely to be discharged home.

Previous studies of community-dwelling middle-aged and older adults have found that being overweight or having low-grade obesity is associated with lower overall mortality in frail individuals, while this benefit was less apparent in nonfrail individuals.^9^ Population studies across various age groups, accounting for underlying disease, indicate that all-cause mortality is lowest within the normal BMI range of 18.5 to 24.9. However, for older adults, particularly septuagenarians and octogenarians, the BMI associated with the lowest mortality tends to be higher.^7,8,9^ Major surgery represents a significant physiological stressor for older adults, particularly for those who are frail and have limited reserves to withstand such stress.^12,24,25,26^ Our findings suggest that all older adults, regardless of frailty status, may benefit from slightly higher BMIs, likely due to the increased demands of surgical recovery. One possible causal mechanism for this observation is that a higher BMI may provide additional energy reserves that are critical for recovery following the acute insult of surgery.^27^ Alternatively, we cannot rule out the potential impact of unmeasured confounding: it is also possible that having a higher BMI is simply a marker of better overall health; many patients in the normal BMI category may have had occult underlying illness that was not captured by our models and put them at a higher risk of mortality. It is also possible that older adults should be categorized into different BMI zones to better capture the risk profile of older adults undergoing surgery.

A significant proportion of patients in the normal BMI range experienced adverse postoperative events, including all-cause mortality and other complications. To investigate this further as a hypothesis-generating subanalysis, we performed a sensitivity and hypothesis-generating analysis by subdividing the normal BMI category into 2 subgroups: 18.5 to 22.5 and 22.5 to 24.9, based on a Youden index cutoff of BMI 22.5. Nearly all mortality and complication events occurred in the lower subcategory (18.5-22.5), reinforcing the concept of a protective role of a slightly higher BMI in older adults undergoing major surgery.

It is important to note, however, that the benefits of being overweight or having low-grade obesity do not extend to individuals with the highest BMIs; those with high-grade obesity experienced significantly higher rates of postoperative complications, excluding all-cause mortality. Specifically, patients with BMIs higher than 40.0 had the highest complication rate, with 79% experiencing at least 1 complication. Pulmonary complications were most prevalent in this group, with 33% requiring reintubation, an upgrade in care, or developing pneumonia during their hospitalization. Additionally, these patients were more likely to develop DVTs or experience concerns for a stroke. Conversely, underweight patients (BMI <18.5) had the highest risk of all-cause mortality, likely reflecting diminished physiological reserves and increased vulnerability to the acute stress of surgery.

An obesity paradox has previously been described and refers to the counterintuitive observation that, in certain populations and clinical conditions, individuals with overweight or mild obesity (typically defined as BMI 25.0-35.0) sometimes have better survival outcomes than those with a normal body mass index (BMI 18.5-24.9).^28^ Possible explanations for this include increased nutritional and metabolic reserves with more adipose and muscle mass that may help withstand catabolic stress, such as during illness, surgery, or hospitalization.^28,29^ Another explanation may be lead-time bias where some patients with lower BMI may have undiagnosed chronic illnesses causing weight loss before diagnosis.^29^ However, the obesity paradox does not imply that obesity is protective overall, rather, it highlights the complexity of how weight, fat distribution, and muscle mass interact with specific disease processes and outcomes.^30^

### Limitations

This study has several limitations. First, the single-center, observational design limits the generalizability of the findings and leaves room for potential confounding variables that were not accounted for in the analysis, such as the example of occult illness mentioned previously. One of the most significant limitations is that BMI does not differentiate between adipose tissue distribution, such as visceral or abdominal fat, which is strongly associated with metabolic syndrome and related complications. Moreover, BMI does not account for variations in body composition, such as differences in muscle mass and fat distribution, which can differ substantially across genders, races, and ethnicities. Indeed, previous studies have shown that BMI alone may be an inadequate measure of health or surgical risk in diverse populations.^31^ BMI was never intended to serve as a single marker of postoperative risk. Additionally, the mFI-5 is determined as a composite of comorbidity and does not evaluate other items of frailty including nutritional status. This may account for the seemingly improved outcomes in prefrail older adults. Furthermore, the sample sizes in the underweight and morbidly obese BMI categories are small, and the resulting 95% CIs are wide; the lower bounds of these intervals remain far from 1, suggesting potentially meaningful associations despite limited precision.

Further research is needed to replicate these findings in larger, multicenter studies and ultimately clinical trials to determine when if ever weight loss should be recommended preoperatively for older adults. Future investigations should also explore how BMI interacts with other critical factors, including nutritional status, muscle mass, and frailty, to refine preoperative optimization strategies. Tailoring these strategies to the unique needs of older adults might improve surgical outcomes and reduce complications in this growing population.

## Conclusions

In this study, older adults with overweight or low-grade obesity who underwent elective surgery had lower odds of 30-day and 1-year mortality compared with those with a normal BMI. These findings challenge traditional preoperative guidelines emphasizing achievement of normal weight before surgery for older adults.

## Notes

Supplement 1. **eTable 1. **Correlation by BMI and Frailty **eTable 2. **All-Cause Mortality by Body Mass Index With Ideal Body Weight Subdivided at Youden Index Cut-off of BMI 22.5 **eTable 3. **Detailed Complications by Body Mass Index **eTable 4. **Complications by Frailty

Supplement 2.
Data Sharing Statement
