# Error in Conclusions

**DOI:** 10.1001/jamanetworkopen.2025.41245

**Published:** 2025-10-08

In the Original Investigation titled “Body Mass Index and Postsurgical Outcomes in Older Adults,”^1^ published August 26, 2025, there were errors in the Conclusions. The Conclusions should have stated that older adults with overweight or low-grade obesity who underwent elective surgery had lower odds of 30-day and 1-year mortality compared with those with a normal BMI, not higher odds. This article has been corrected.^1^
