# Supplementary material for: Body Mass Index and Postsurgical Outcomes in Older Adults
Source: JAMA Netw Open. 2025 Aug 26;8(8):e2528875. doi: 10.1001/jamanetworkopen.2025.28875 (PMC12381675; doi:10.1001/jamanetworkopen.2025.28875)
Supplement: Supplement 1. — eTable 1. Correlation by BMI and Frailty eTable 2. All-Cause Mortality by Body Mass Index With Ideal Body Weight Subdivided at Youden Index Cut-off of BMI 22.5 eTable 3. Detailed Complications by Body Mass Index eTable 4. Complications by Frailty [file jamanetwopen-e2528875-s001.pdf]

Supplemental Online Content

Canales C, Anderson M, Elashoff D, et al. Body mass index and postsurgical outcomes in older adults. *JAMA Netw Open*. 2025;8(8):e2528875. doi:10.1001/jamanetworkopen.2025.28875

eTable 1. Correlation by BMI and Frailty
eTable 2. All-Cause Mortality by Body Mass Index With Ideal Body Weight Subdivided at Youden Index Cut-off of BMI 22.5
eTable 3. Detailed Complications by Body Mass Index
eTable 4. Complications by Frailty

This supplemental material has been provided by the authors to give readers additional information about their work.

© 2025 Canales C et al. *JAMA Network Open*.

eTable 1. Correlation by BMI and Frailty

BMI Category	Frailty Groups Not Frail (n=161)	Pre-Frail (n=153)	Frail (n=100)
**Underweight**	7 (4.3%)	9 (5.9%)	4 (4.0%)
**Healthy Weight**	61 (37.9%)	41 (26.8%)	31 (31.0%)
**Overweight**	41 (25.5%)	56 (36.6%)	31 (31.0%)
**Obese**	46 (28.6%)	40 (26.1%)	23 (23.0%)
**Morbid Obese**	6 (3.7%)	7 (4.6%)	11 (11.0%)

Spearman correlation r=0.06, p=0.24

© 2025 Canales C et al. *JAMA Network Open*.

eTable 2. All-Cause Mortality by Body Mass Index With Ideal Body Weight Subdivided at Youden Index Cut-off of BMI 22.5 Kg/m^2

	Body Mass Index Categories (Kg/m^2)						
	< 18.5 (n=20)	18.5-22.49 (n=51)	22.5-24.9 (n=12)	25-29.9 (n=128)	30-39 (n=109)	40> (n=24)	p-value chi-square
30 Day Mortality	15 (75.0%)	23 (37.1%)	2 (2.8%)	1 (0.8%)	1 (0.9%)	5 (20.8%)	<0.001
1-year Mortality	16 (80.0%)	28 (45.2%)	10 (14.1%)	7 (5.5%)	5 (4.6%)	6 (25.0%)	<0.001

© 2025 Canales C et al. *JAMA Network Open*.

eTable 3. Detailed Complications by Body Mass Index

	Body Mass Index Categories (Kg/m^2)					
	< 18.5 (n=20)	18.5-24.9 (n=133)	25-29.9 (n=128)	30-39 (n=109)	40> (n=24)	p-value chi-square
30 Day Mortality	15 (75.0%)	25 (18.8%)	1 (0.8%)	1 (0.9%)	5 (20.8%)	<0.001
1-year Mortality	16 (80.0%)	38 (28.6%)	7 (5.5%)	5 (4.6%)	6 (25.0%)	<0.001
Delirium	10 (50.0%)	19 (14.3%)	14 (10.9%)	14 (12.8%)	5 (20.8%)	0.001
Discharge other than Home	10 (50.0%)	21 (15.8%)	9 (7.0%)	16 (14.7%)	7 (29.2%)	<0.001
Reoperation	0	3 (2.3%)	0	1 (0.9%)	2 (8.3%)	0.043
30 Day Readmission	0	1 (0.8%)	0	1 (0.9%)	3 (12.5%)	0.003
Cardiac Complication	1 (5.0%)	10 (7.5%)	2 (1.6%)	6 (5.5%)	2 (8.3%)	0.126
Pulmonary Complications	1 (5.0%)	3 (2.3%)	0	2 (1.8%)	8 (33.3%)	<0.001
Upgrade in Care	0	4 (3.0%)	0	0	3 (12.5%)	0.001
Other Complications	0	3 (2.3%)	2 (1.6%)	1 (0.9%)	5 (20.8%)	<0.001
Any Complication	13 (65.0%)	36 (27.1%)	20 (15.6%)	19 (17.4%)	19 (79.2%)	<0.001
Clavien-Dindo Classification						<0.001
0	6 (30.0%)	90 (67.7%)	107 (83.6%)	90 (82.6%)	5 (20.8%)	
1	2 (10.0%)	7 (5.3%)	14 (10.9%)	5 (4.6%)	4 (16.7%)	
2	1 (5.0%)	12 (9.0%)	6 (4.7%)	12 (11.0%)	8 (33.3%)	
3	0	1 (0.8%)	0	1 (0.9%)	1 (4.2%)	
4	0	3 (2.3%)	0	0	2 (8.3%)	
5	11 (55.0%)	20 (15.0%)	1 (0.8%)	1 (0.9%)	4 (16.7%)	

© 2025 Canales C et al. *JAMA Network Open*.

eTable 4. Complications by Frailty

	Not Frail (n=161)	Pre-Frail (n=153)	Frail (n=100)	P-value
30 Day Mortality	16 (9.9%)	10 (6.5%)	21 (21.0%)	0.001
1-year Mortality	25 (15.5%)	18 (11.8%)	29 (29.0%)	0.001
Delirium	18 (11.2%)	22 (14.4%)	22 (22.0%)	0.057
Discharge other than Home	18 (11.2%)	23 (15.0%)	22 (22.0%)	0.061
Reoperation	4 (2.5%)	1 (0.7%)	1 (1.0%)	0.516
30 Day Readmission	1 (0.6%)	2 (1.3%)	2 (2.0%)	0.550
Cardiac Complication	4 (2.5%)	1 (0.7%)	16 (16.0%)	<0.001
Pulmonary Complications	3 (1.9%)	2 (1.3%)	9 (9.0%)	0.004
Upgrade in Care	3 (1.9%)	0	4 (4.0%)	0.041
Other Complications	2 (1.2%)	1 (0.7%)	8 (8.0%)	0.002
Any Complication	37 (23.0%)	30 (19.6%)	40 (40.0%)	<0.001
Clavien-Dindo Classification				<0.001
0	123 (76.4%)	119 (77.8%)	56 (56.0%)	
1	14 (8.7%)	14 (9.2%)	4 (4.0%)	
2	8 (5.0%)	11 (7.2%)	20 (20.0%)	
3	2 (1.2%)	1 (0.7%)	0	
4	2 (1.2%)	0	3 (3.0%)	
5	12 (7.5%)	8 (5.2%)	17 (17.0%)	

© 2025 Canales C et al. *JAMA Network Open*.
