# Supplementary material for: Body Mass Index and Postsurgical Outcomes in Older Adults
Source: JAMA Netw Open. 2025 Aug 26;8(8):e2528875. doi: 10.1001/jamanetworkopen.2025.28875 (PMC12381675; doi:10.1001/jamanetworkopen.2025.28875)
Supplement: Supplement 2. — Data Sharing Statement [file jamanetwopen-e2528875-s002.pdf]

Data Sharing Statement

Canales. Body Mass Index and Postsurgical Outcomes in Older Adults. *JAMA Netw Open*. Published August 26, 2025. doi:10.1001/jamanetworkopen.2025.28875

Data
Data available: No

Additional Information
Explanation for why data not available: Will be shared with IRB approval upon request
